# Genetic variation in NRG 1 gene and risk of post‐traumatic stress disorders in patients with hepatocellular carcinoma

**DOI:** 10.1002/jcla.23187

**Published:** 2020-01-15

**Authors:** Liumei Luo, Li Li, Min Guo, Xi Chen, Yuzhu Lin, Dingyin Wu

**Affiliations:** ^1^ Xiangya Nursing College Central South University Changsha China; ^2^ Department of science and education Hainan General Hospital Haikou China; ^3^ Department of nursing Xiangya Medical College of Central South University Changsha China

**Keywords:** hepatocellular carcinoma, NRG1, post‐traumatic stress disorders, rs35753505, rs3924999

## Abstract

**Objective:**

Neuregulin 1 (NRG1) was proved to play an important role in numerous neurodevelopmental processes. In our study, we aimed to investigate the relationship between the NRG1 gene polymorphism and the cognitive function of patients with hepatocellular carcinoma (HCC) complicated with post‐traumatic stress disorders (PTSD) before and after the psychological intervention.

**Methods:**

Mini‐mental State Examination (MMSE), Montreal Cognitive Assessment (MoCA), and Loewenstein Occupational Therapy Cognitive Assessment (LOTCA) were used for cognitive function assessment. Serum level of NRG1 was detected by ELISA, and the correlation between NRG1 level and cognitive function was analyzed. The difference of cognitive function score of patients with HCC complicated with PTSD before and after psychological intervention was compared, and the relationship between rs35753505 and rs3924999 polymorphism with the score was analyzed.

**Results:**

Patients with HCC complicated with PTSD showed decreased serum NRG1 level. NRG1 levels of patients in the HCC + PTSD group were positively correlated with MMSE, MoCA, and LOTCA scores. In rs35753505, the CC genotype was a risk factor for the occurrence of PTSD in patients with HCC, while in rs3924999, the GG genotype was a risk factor for the occurrence of PTSD in patients with HCC. After psychological intervention, the CC genotype at rs35753505 and the GG genotype at rs3924999 were susceptible genotypes.

**Conclusion:**

CC genotype at rs35753505 and GG genotype at rs3924999 of NRG1 gene increased the risk of PTSD in patients with HCC. CC and GG genotypes were susceptible after psychological intervention.

## 1 INTRODUCTION

Hepatocellular carcinoma (HCC) is the sixth most common cancer around the world and the second leading cause of cancer death.^1^ Statistics show that the incidence of HCC is particularly high in eastern/south‐eastern Asia and Africa, intermediate in Southern Europe, and low in most high‐income countries.^2^ To be specific, the average age of diagnosis is 65 and has gradually shifted to early diagnosis over the past decade, and males are more likely to suffer HCC than females.^3^ It is reported that patients with HCC has low 5‐year survival rate (~16%) partly due to the lack of effective therapeutic methods.^4^ Thus, it is very urgent to find new therapeutic methods for HCC treatment. Post‐traumatic stress disorder (PTSD) is a common severe traumatic mental disorder which can show itself in multiple kinds of ways such as a persistent re‐experiencing of the traumatic event, numbing, avoidance, fear, and/or startle responses.^5^, ^6^ Trauma exposure is an important factor in the diagnosis of PTSD, including physical or sexual abuse, natural or man‐made disasters, witnessing domestic violence, catastrophic illnesses, war or terrorism, vehicle or other accidents.^7^ Importantly, life‐threatening illness has also been regarded as a stressor that can elicit PTSD.^8^ Psychotherapy was reported as the main treatment method to address PTSD symptoms.^7^ However, the mechanisms underlying HCC patients complicated with PTSD are not yet fully understood.

Neuregulin 1 (NRG1) is a trophic factor that works via the activation of ErbB receptor tyrosine kinases, including ErbB4.^9^ NRG1 signaling has been reported to be involved in many kinds of steps in neural development, including synapse formation, axon guidance, neuron migration, and expression of neurotransmitter receptors.^10^ Besides, NRG1 can also affect neuronal plasticity and the organization of neuronal networks in the brain.^11^ Accumulating evidence has demonstrated that the single‐nucleotide polymorphisms (SNPs) of NRG1 SNP8NRG221533 (rs35753505), SNP8NRG241930 (rs62510682), and SNP8NRG243177 (rs6994992) are related to some neuropsychiatric disorders, including schizophrenia and major depression.^12^, ^13^ Besides, previous evidence has also pointed out that rs3924999 in NRG1 gene might affect cognitive processing functionally.^14^ Here in this study, we attempted to address the correlation between the NRG1 gene polymorphism (rs35753505 and rs3924999) and the cognitive function of the patients with HCC complicated with PTSD before and after the psychological intervention.

## 2 MATERIALS AND METHODS

### 2.1 Ethical statement

This study was approved and supervised by the Ethics Committee of Hainan General Hospital. All procedures in the present study were performed in accordance with the ethical code of the Ethics Committee of Hainan General Hospital with (2019) No. 15 and in accordance with the Helsinki Declaration (the 2013 revision). Signed informed consent was obtained from each eligible participant or their family members.

### 2.2 Study subjects

The sample size was evaluated as per the formula: N = (Uα/δ)^2^ (1‐P) P, in which the parameter α = 0.05, β = 0.20, and δ = 0.08. P sensitivity =0.75, and P specificity =0.55. (P sensitivity was used when measuring the sample size of the experimental group, while P specificity was used when measuring the sample size of the control group). A total of 234 HCC patients diagnosed in our hospital from March 2017 to August 2018 were included. Among them, there were 101 HCC patients complicated with PTSD (male: 61; female: 40; mean age: 64.0 ± 7.5), and 133 HCC patients not complicated with PTSD (male: 79; female: 54; mean age: 65.5 ± 8.2). The inclusion criteria were as follows: (a) All patients were diagnosed as primary HCC by pathology and clinical diagnosis; (b) patients with HCC complicated with PTSD were diagnosed by two psychiatrists of intermediate professional titles or above with abundant clinical experience and diagnosed in accordance with the unified diagnostic criteria (DSM‐IV, PTSD)^15^; (c) patients had no other serious physical diseases and felt good in physical condition. The exclusion criteria were as follows: (a) Patients with secondary malignant HCC or recurrent HCC; (b) patients complicated with other tumors or wasting diseases; (c) patients had a family history of genetic psychosis. At the same time, 148 healthy volunteers (male: 101; female: 47; mean age: 62.0 ± 7.3) who underwent routine physical examination in our hospital during the same period were recruited as the control group.

### 2.3 Psychological interventions

On the basis of establishing good nurse‐patient relationship, we strengthened communication with patients, and implemented early psychological nursing and intervention. We contacted the patient's family, friends, and leaders in work to bring them strong support from the family and society. Great efforts were made in pain management to reduce anxiety, depression, and other adverse emotions, to improve the quality of life, and to promote the relief and elimination of PTSD symptoms of patients. PDCA cycle was applied. In detail, this cycle includes plan (P), do (D), check (C), and action (A), which is one of the most common nursing models to improve the quality of nursing as previously reported.^16^ Health education was implemented to increase patients' knowledge of disease, so that they could positively cooperate with medical and nursing staffs for better recovery. Besides, continuous guidance in rehabilitation was performed to promote the early recovery of patients, to have the patients returned to their social roles as soon as possible, thus to promote the comprehensive physical and mental recovery of patients.

### 2.4 Cognitive function test

Cognitive function of all subjects was evaluated via Mini‐mental State Examination (MMSE).^17^ MMSE provides a measure of orientation, memory, recall, and language ability with a total score of 0‐30. The score of cognitive function was the sum of all correctly answered small items, with a total of 30 items and one score for each. Patients scored at 1‐24 were regarded as cognitively impaired. The retest reliability was 0.80‐0.99, and the reliability among the subjects was 0.95‐1.00.

Montreal Cognitive Assessment scale (MoCA) includes eight cognitive fields including attention and concentration, executive function, memory, language, visual structure skills, abstract thinking, calculation, and orientation. A total of 11 items were included with a total score of 30 points. More than 26 scores were considered as normal values, and the instant memory was not counted. The subjects whose cultural and educational level is <12 years were added with one extra score to balance the bias of the cultural level. The higher scores indicate better cognitive performances. Each test was completed within 10 minutes, with the score of 26 points was set as the cutoff value.

Loewenstein Occupational Therapy Cognitive Assessment (LOTCA)^18^ includes five aspects (20 items): orientation, perception, visual motor organization, thinking operation, and attention. The score of concentration was determined based on the time spent and the reminding times in the test. The lowest score for each item was 1 point while the full score was 4‐5 points (the first three sub‐items in the operation of thinking adopt five‐point scale, while the rest items adopt of four‐point scale), and the total score for all items was 91 points. According to the requirements of the scale, the evaluation of all cases was completed in a standard cognitive function assessment room.

### 2.5 Enzyme‐linked immunosorbent assay (ELISA)

The blood of the elbow vein was extracted from fasting patients in the morning, slowly injected into non‐anticoagulant tubes, allowed to stand at room temperature for 60 minutes, and centrifuged at 1000 r/min for 20 minutes. The serum was extracted and placed in Eppendorf tubes and then stored at −80°C. The level of NRG1 was determined according to the instructions of ELISA kit (R&D Systems).

### 2.6 Genotyping

Peripheral venous blood (5 mL) was collected from fasting patients in the morning, anticoagulated by ethylene diamine tetraacetic acid and stored at −80°C for the preparation of genomic DNA. Total genomic DNA was extracted by selective adsorption DNA kit (Corning Life Science) through cell lysis and heme/protein precipitation techniques. Next, 1 μL DAN sample was run on 1% agarose gel electrophoresis to assess the quality of all DNA samples. The DNA concentration of the samples was estimated according to the brightness of the imaging bands on an ultraviolet imager, and the samples were diluted to the working concentration 10‐20 ng/μL.

The NRG1 gene sequence (NC_000008.11) was searched from the National Center for Biotechnology Information (NCBI). The primers were designed using Premier printer5 software and had the coding sequence and shear site sequence amplified. The primers were synthesized by Sangon Biotech Co., Ltd. The primers of rs35753505 were 5′GAGACTGGAAGCCATGTATC‐3′ (forward) and 5′GGCATCAGTTTTCAATAGC‐3′ (reverse); the primers of rs3924999 were 5′ACTGGTTTCACACCGAAGGAC‐3′ (forward) and 5′CCAAGATGAGATCCATTTTCGC‐3′ (reverse). The PCR system (25 µL) consisted of 12.5 µL 2 × PCR Master Mix, 1 µL upstream and 1 µL downstream primers, 2 µL DNA templates and 8.5 µL nuclease free water, and the reaction system was pre‐denaturation at 95°C for 3 minutes, and a total of 35 cycles of denaturation at 94°C for 30 seconds, annealing at 58°C for 30 seconds, extension at 72°C for 30‐60 seconds, and a final extension at 72°C for 10 minutes. The PCR product was identified as the target product following 1.5% agarose gel electrophoresis analysis. After purification of PCR products, ABI PRISM3730XL automatic sequencers were used to sequence the products. The sequencing was completed by Tsing Ke Biological Technology. Sequencer4.10.1 demo software was used to compare and analyze the genome sequences to find out if there was any mutation.

### 2.7 Statistical analysis

All statistical analyses were performed using SPSS version 21.0 (IBM Corp.). Measurement data were expressed as mean ± standard deviation. The *t* test was applied for comparisons between two groups, while one‐way analysis of variance (ANOVA) for multiple groups and Tukey's multiple comparisons test for pair‐wise comparisons after ANOVA analysis. Enumeration data were expressed as (n [%]) and tested by Fisher's exact test. Correlation between NRG1 level and the cognitive function of HCC complicated with PTSD patients was analyzed using Pearson correlation analysis. *P* value was obtained by two‐tailed test, and *P* < .05 was regarded to show significant difference.

## 3 RESULTS

### 3.1 Comparison of the baseline characteristics

There was no significant difference in the mean age, gender, and body mass index among the control, HCC and HCC + PTSD groups (all *P* > .05). Patients in the HCC and HCC + PTSD groups had higher rates of smoking history, drinking history, and hepatitis B when compared to those in the control group (all *P < *.001; Table 1).

**Table 1:** Comparisons of baseline characteristics among three groups

	Control group	HCC group	HCC + PTSD group (n = 101)	*P* *	*P* **	*P* †
	(n = 148)	(n = 133)				
Mean age (y)	62.0 ± 7.3	65.5 ± 8.2	64.0 ± 7.5	.136	.657	.364
Gender (male/female)	101/47	79/54	61/40	.224	.894	.249
Smoking history (yes/no)	45/103	79/54	61/40	**<.05**	**<.05**	.894
Drinking history (yes/no)	53/95	71/62	59/42	**<.05**	**<.05**	.507
BMI index (kg/m^2^)	23.76 ± 3.1	24.01 ± 4.3	23.15 ± 3.3	.582	.143	.104
Hepatitis B (yes/no)	19/129	77/54	63/38	**<.001**	**<.001**	.591

Note

The data were tested by *t* test.

* The HCC group vs the control group.

** The HCC + PTSD group vs the control group.

^†^ The HCC + PTSD group vs the HCC group.

All the *P* values in bold are less than 0.05 that represent significant difference.

### 3.2 Patients with HCC complicated with PTSD showed decreased serum NRG1 level

**Figure 1:**
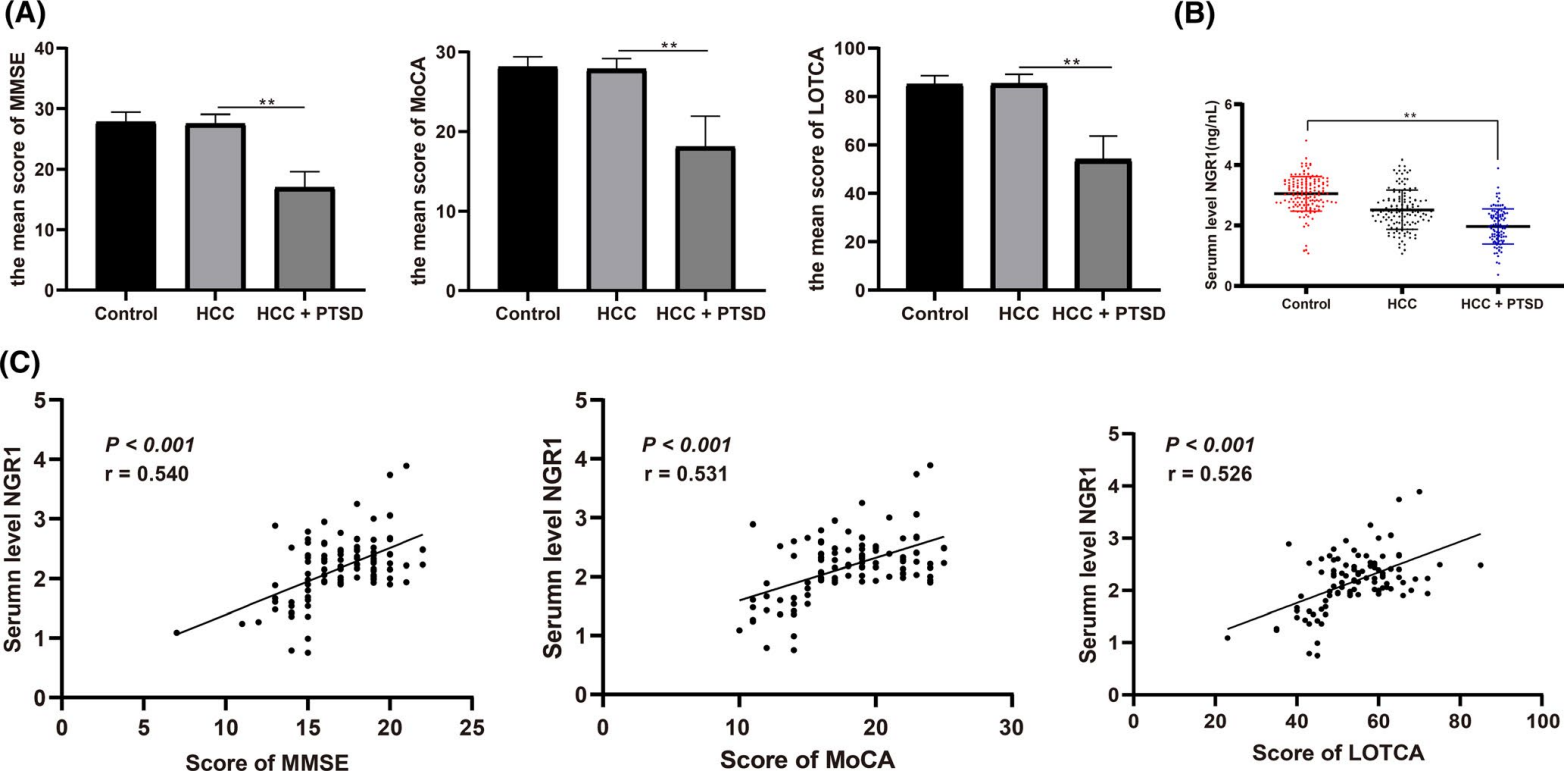
Patients with HCC complicated with PTSD showed decreased serum NRG1 level. A, Mean score of cognitive function in each group; B, serum NRG1 level of patients was detected by ELISA; C, Correlation between serum NRG1 level and cognitive function in patients with HCC complicated with PTSD; control group, n = 148, HCC group, n = 133, HCC + PTSD group, n = 101; in panel A and B, data were analyzed using one‐way analysis of variance and Tukey's multiple comparisons test; in panel C, Pearson correlation analysis was conducted. **P < .05

Cognitive function of the study subjects was scored by the MMSE, MoCA, and LOTCA scales. It was found that the cognitive function scores of patients in the HCC + PTSD group were significantly lower than that of the participants in the control and the HCC groups (*P* < .05; Figure 1A). The NRG1 gene is the susceptible gene of schizophrenia.^19^ We discussed the role of NRG1 gene in the patients with HCC complicated with PTSD, and it was found that compared to the control group, there was no significant difference in the NRG1 level in patients in the HCC group (*P* > .05), while significant decreased NRG1 level was found in patients in the HCC + PTSD group (*P* < .05; Figure 1B).

Pearson correlation analysis showed that NRG1 levels of patients in the HCC + PTSD group were positively correlated with MMSE scores (*r* = 0.540, *P* < .001), MoCA scores (*r* = 0.5305, *P* < .001), and LOTCA scores (*r* = 0.5257, *P* < .001; Figure 1C). It is suggested that the serum level of NRG1 in HCC patients is related to the cognitive function of patients with HCC complicated with PTSD.

### 3.3 Genotyping of NRG1 gene polymorphism rs35753505 and rs3924999

**Figure 2:**
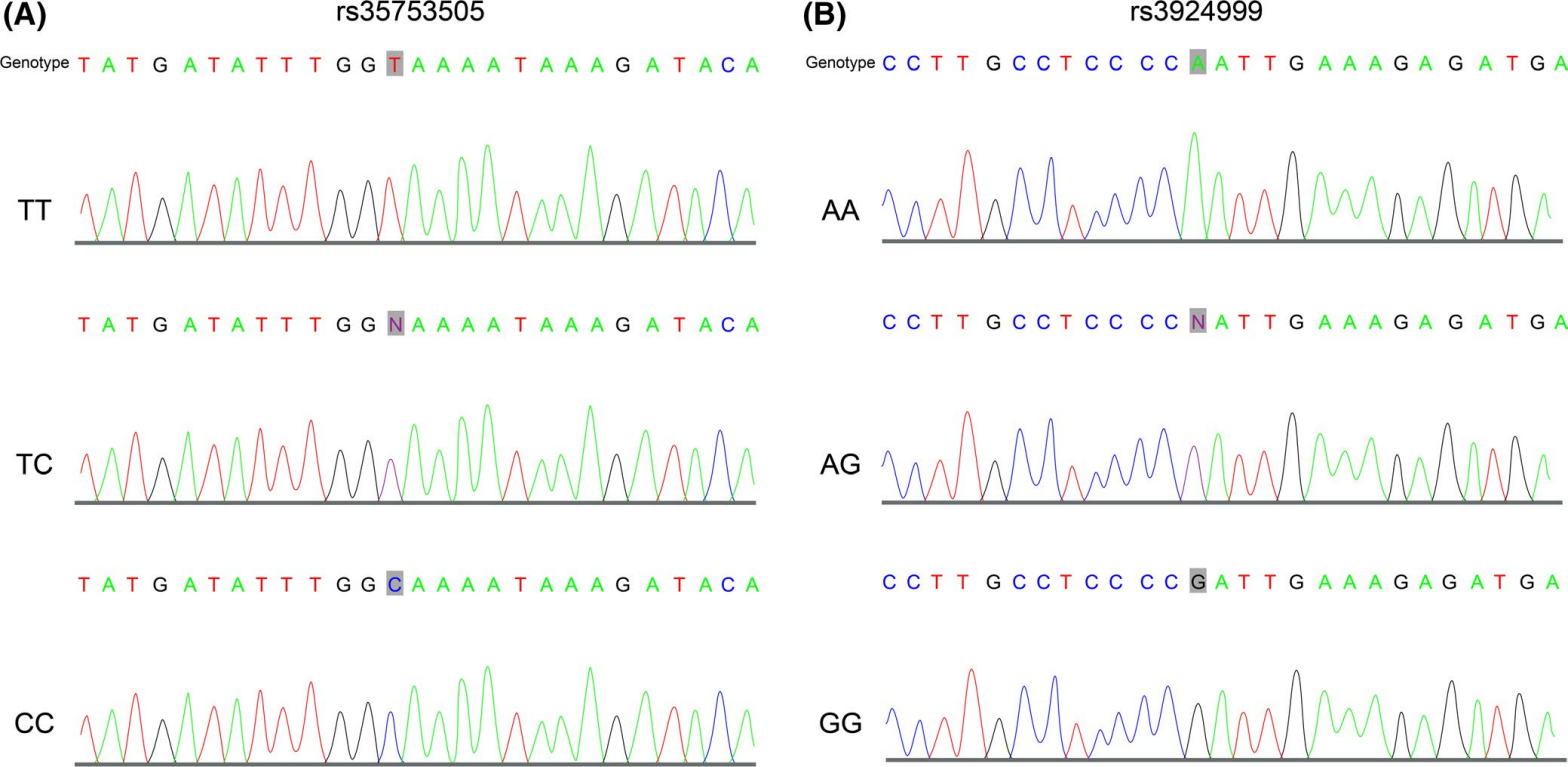
Genotyping of NRG1 gene polymorphism rs35753505 and rs3924999. A, sequencing results of rs35753505; B, sequencing results of rs3924999

rs35753505 of NRG1 plays an important role in conferring susceptibility to the schizophrenia in a Pakistani population.^20^ Besides, alleles at the rs3924999 of the NRG1 gene increase the risk of schizophrenia.^21^ Therefore, we studied the rs35753505 and rs3924999 polymorphism of the NRG1 gene in the occurrence of PTSD in patients with HCC. Three genotypes of rs35753505: TT, TG, and GG (Figure 2A), and the three genotypes of rs3924999: AA, AG and GG (Figure 2B) were found by sequencing.

### 3.4 CC genotype of rs35753505 and GG genotype of rs3924999 were risk factors of PTSD in HCC patients

The genotype distribution was analyzed and the genotypes were subjected to Hardy‐Weinberg equilibrium analysis. The results showed that the genotype distribution of rs35753505 and rs3924999 in the NRG1 gene in the control group was in accordance with the Hardy‐Weinberg equilibrium (*P* = .533, *P* = .340), suggesting the population representativeness. In addition, the successful rate of genotyping of rs35753505 and rs3924999 was 100%.

There was no significant difference in the allelic and genotypic distribution of rs35753505 and rs3924999 between the control group and the HCC group (*P* > .05). Significant difference was found in comparisons of CC genotypic distribution of rs35753505 and GG genotypic distribution of rs3924999 between the HCC and HCC + PTSD groups (*P* < .05). At rs35753505, compared to the TT genotype, the CC genotype was a risk factor for the occurrence of PTSD in patients with HCC (OR = 2.077, 95% CI = 1.066‐4.141, *P* = .041); while the C allele was a risk factor compared to the T allele (OR = 1.602, 95%CI = 1.106‐2.296, *P* = .015). At rs3924999, compared to the AA genotype, the GG genotype was a risk factor for the occurrence of PTSD in patients with HCC (OR = 2.126, 95% CI = 1.045‐4.222, *P* = .040); the G allele was a risk factor compared to the A allele (OR = 0.551, 95% CI = 0.3774‐0.807, *P* = .003; Tables 2 and 3).

**Table 2:** Comparison of genotype distribution of rs35753505 and rs3924999 in the NRG1 gene between the control and HCC groups

SNP	Control group (n = 148)	HCC group (n = 133)	*P*	OR (95%CI)
rs35753505				
TT	43 (29.05)	36 (24.32)		
TC	70 (47.30)	67 (45.27)	.673	1.143 (0.6533‐2.022)
CC	35 (23.65)	30 (20.27)	>.9999	1.024 (0.5170‐2.021)
TC + CC	105 (70.95)	97 (72.93)	.791	1.103 (0.6498‐1.828)
T	156 (52.7)	139 (52.26)	.933	1.018 (0.7323‐1.415)
C	140 (47.30)	127 (47.74)		
rs3924999				
AA	36 (24.32)	32 (21.62)		
AG	68 (45.95)	65 (43.92)	.882	1.075 (0.6027‐1.935)
GG	44 (29.73)	36 (24.32)	.869	0.921 (0.4753‐1.781)
AG + GG	104 (70.27)	101 (75.94)	.781	1.093 (0.6301‐1.915)
A	129 (48.50)	69 (34.16)	.8	1.049 (0.7550‐1.459）
G	137 (51.50)	133 (65.84)		

Note

The data were analyzed by Fisher's exact test.

Abbreviations: CI, confidence interval; OR, odds ratio.

**Table 3:** Comparison of genotype distribution of rs35753505 and rs3924999 in the NRG1 gene between the HCC and HCC + PTSD groups

SNP	HCC (n = 133)	HCC + PTSD (n = 101)	*P*	OR (95%CI)
rs35753505				
TT	36 (27.07)	26 (25.74)		
TC	67 (50.38)	30 (29.70)	.176	0.620 (0.3293‐1.189)
CC	30 (22.56)	45 (44.55)	**.041** *	2.077 (1.066‐4.141)
TC + CC	97 (72.93)	75 (74.26)	.882	1.071 (0.6018‐1.965)
T	139 (52.26)	82 (40.59)	**.015** **	1.602 (1.106‐2.296)
C	127 (47.74)	120 (59.41)		
rs3924999				
AA	32 (31.68)	23 (22.77)		
AG	65 (64.36)	23 (22.77)	.066	0.492 (0.2498‐1.028)
GG	36 (35.64)	55 (54.46)	**.040** *	2.126 (1.045‐4.222)
AG + GG	101 (75.94)	78 (77.23)	.877	1.074 (0.5758‐1.978)
A	129 (48.50)	69 (34.16)	**.003** **	0.551 (0.3774‐0.807)
G	137 (51.50)	133 (65.84)		

Note

The data was analyzed by Fisher's exact test.

Abbreviations: OR, odds ratio; CI, confidence interval.

* Compared with genotype in the HCC group, *P* < .05.

** Compared with allele in the HCC group, *P* < .05.

All the *P* values in bold are less than 0.05 that represent significant difference.

### 3.5 Relationship between NRG1 gene polymorphism and cognitive function of patients with HCC complicated with PTSD before and after psychological interventions

The scores of cognitive function of patients with HCC complicated with PTSD before and after psychological intervention were statistically analyzed. It was found that the score of cognitive function increased significantly after psychological interventions (*P* < .05). The relationship between rs35753505 and rs3924999 polymorphisms and cognitive function scores of patients with HCC complicated with PTSD before and after psychological interventions was analyzed. The results found that at rs35753505, compared with the TT genotype, the scores of MMSE, MoCA, and LOTCA in the CC genotype of patients with HCC complicated with PTSD were significantly higher (*P* < .05). At rs3924999, compared with the AA genotype, the score of cognitive function in the GG genotype of patients with HCC complicated with PTSD was significantly higher after psychological intervention (*P* < .05; Tables 4,5, and 6). After psychological intervention, the CC genotype at rs35753505 and the GG genotype at rs3924999 were susceptible genotypes, which had an important effect on cognitive function of patients with HCC complicated with PTSD.

**Table 4:** Comparison of MMSE scores before and after psychological intervention in patients with HCC complicated with PTSD of different genotypes

SNP	HCC + PTSD	before	after	Difference	*t*	*P*
rs35753505						
TT	26	18.67 ± 1.47	21.31 ± 0.77*	2.00 ± 1.59		
TC	30	19.0 1 ± 1.53	21.72 ± 0.45*	2.71 ± 1.69	0.31	.756
CC	45	14.69 ± 1.81	23.4 ± 0.68**	8.71 ± 2.43	11.23	<.0001
rs3924999						
AA	23	19.33 ± 1.27	21.43 ± 0.71*	2.11 ± 1.55		
AG	23	19.31 ± 1.25	21.43 ± 0.65*	2.12 ± 1.32	0.041	.967
GG	55	15.04 ± 1.77	23.16 ± 0.80**	8.12 ± 2.53	10.47	**<.0001**

Note

The data were analyzed by independent sample *t* test.

* Compared with the data before psychological intervention, *P* < .05.

** Compared with the data after psychological intervention, *P* < .001.

All the *P* values in bold are less than 0.05 that represent significant difference.

**Table 5:** Comparison of MoCA scores before and after psychological intervention in patients with HCC complicated with PTSD of different genotypes

SNP	HCC + PTSD	Before	After	Difference	*t*	*P*
rs35753505						
TT	26	20.96 ± 2.17	26.04 ± 4.27*	5.07 ± 4.75		
TC	30	21.06 ± 2.33	26.03 ± 3.08*	4.97 ± 3.93	0.04	.971
CC	45	14.36 ± 1.98	25.33 ± 3.03**	10.98 ± 3.47	5.93	**<.0001**
rs3924999						
AA	23	20.87 ± 2.09	26.70 ± 3.95*	5.83 ± 4.56		
AG	23	21.09 ± 2.47	25.78 ± 3.68*	4.70 ± 4.41	0.84	.408
GG	55	15.53 ± 3.22	25.25 ± 2.93**	9.73 ± 4.27	3.56	**.0006**

Note

The data were analyzed by independent sample *t* test.

* Compared with the data before psychological intervention, *P* < .05.

** Compared with the data after psychological intervention, *P* < .001.

All the *P* values in bold are less than 0.05 that represent significant difference.

**Table 6:** Comparison of LOTCA scores before and after psychological intervention in patients with HCC complicated with PTSD of different genotypes

SNP	HCC + PTSD	Before	After	Difference	*t*	*P*
rs35753505						
TT	26	58.34 ± 8.37	67.54 ± 2.06*	9.19 ± 7.73		
TC	30	57.55 ± 6.46	67.03 ± 2.77*	9.48 ± 7.09	0.14	.892
CC	45	49.44 ± 9.23	64.69 ± 3.84**	12.67 ± 5.54	3.29	**.002**
rs3924999						
AA	23	58.57 ± 8.77	67.57 ± 2.14*	9.00 ± 8.04		
AG	23	57.04 ± 6.60	67.48 ± 2.82*	10.43 ± 7.21	0.62	.536
GG	55	51.04 ± 9.83	64.89 ± 3.59**	13.85 ± 7.44	2.53	**.013**

Note

The data were analyzed by independent sample *t* test.

* Compared with the data before psychological intervention, *P* < .05.

** Compared with the data after psychological intervention, *P* < .001.

All the *P* values in bold are less than 0.05 that represent significant difference.

## 4 DISCUSSION

HCC is famous for its high recurrence rate and poor prognosis, so it is necessary to identify new predictors of HCC and explore the potential mechanisms.^22^ In addition, life‐threatening illnesses, especially cancers, have the potential to lead to PTSD.^23^ Psychotherapy is of great help to patients with mental illness.^24^ In the present study, we aimed to investigate the relationship between the NRG1 gene polymorphism (rs35753505 and rs3924999) and the cognitive function of the patients with HCC complicated with PTSD before and after the psychological intervention.

We firstly performed cognitive function evaluation for all the enrolled subjects, and the results showed that patients with HCC complicated with PTSD had lower cognitive function scores than patients with HCC and healthy controls. Evidence demonstrated that suffering a life‐threatening illness was first thought to be an event that could cause PTSD in the DSM‐IV.^25^ It is reported that PTSD patients have different degrees of cognitive impairment.^26^ Additionally, our study findings suggested that patients with HCC complicated with PTSD had decreased NRG1 level. NRG1, a member of NRG family, is regarded as a major growth factor in the development of the normal nervous system and the occurrence of schizophrenia.^27^ NRG1 is highly expressed in the developing brain.^28^ Previous study noted that restoration of NRG1 could promote oligodendrocyte replacement and white matter repair following spinal cord injury.^29^

Besides, after genotyping, we analyzed the allelic and genotypic distribution of rs35753505 and rs3924999, and the results demonstrated that at the rs35753505, compared to the TT genotype, the CC genotype was a risk factor for the occurrence of PTSD in patients with HCC; the C allele was a risk factor compared to the T allele; in rs3924999, compared to the AA genotype, the GG genotype was a risk factor for the occurrence of PTSD in patients with HCC; the G allele was a risk factor compared to the A allele. NRG1 was reported to attenuate cognitive function impairments in a transgenic mouse model of Alzheimer's disease.^30^ It should be noted that variations in rs35753505 were involved in the microstructure of the medial frontal white matter and abnormalities in the structural connectivity of the right medial temporal lobe in healthy people.^31^, ^32^ The parahippocampal cortex is essential for many higher‐level cognitive procedures, and the medial frontal region is involved in memory/learning.^33^, ^34^ One study focused on bipolar disorder showed that patients carried the C allele of rs35753505 had a greater white matter volume in several regions when compared to those of the TT genotype.^35^ In consistent with our study, He BS et al also proved that the rs3924999 G/G genotype was susceptible to schizophrenia.^21^ Besides, alleles at the rs3924999 of the NRG1 gene increase the risk of schizophrenia.^21^ Furthermore, our study noted that score of cognitive function increased significantly after psychological interventions, and the CC genotype of rs35753505 and the GG genotype of rs3924999 were susceptible genes, which had an important effect on cognitive function of patients with HCC complicated with PTSD. It is reported that psychological interventions can reduce emotional distress for cancer patients.^36^ In addition, previous study also provided evidence that psychological intervention were effective for targeting cancer‐related cognitive decline.^37^

In conclusion, our study findings offer preliminary support that CC genotype at rs35753505 and GG genotype at rs3924999 of NRG1 gene increased the risk of PTSD in patients with HCC. CC genotype and GG genotype were susceptible genes after psychological intervention. This needs to be replicated in larger sample for definitive inference with regard to the interaction between patients with HCC complicated with PTSD effects and NRG1 gene. Further research in this area might promote achievement for addressing PTSD symptoms in HCC.

## Data Availability

All the data generated or analyzed during this study are included in this published article.
